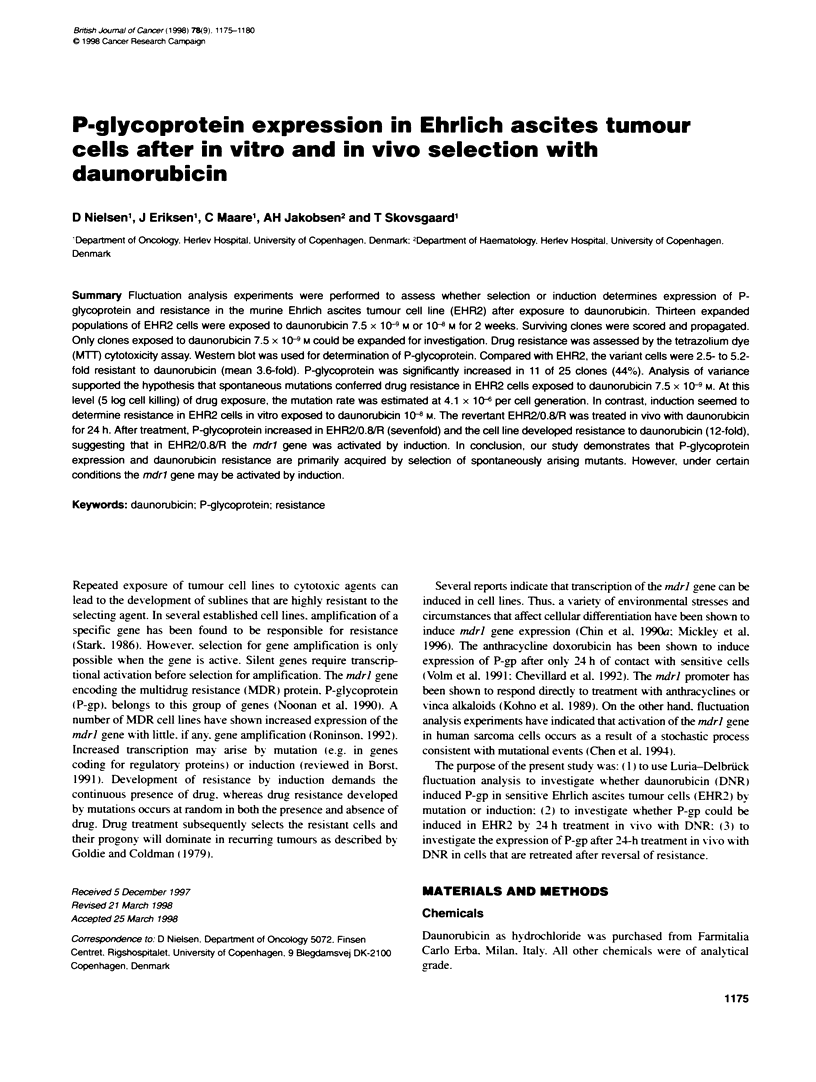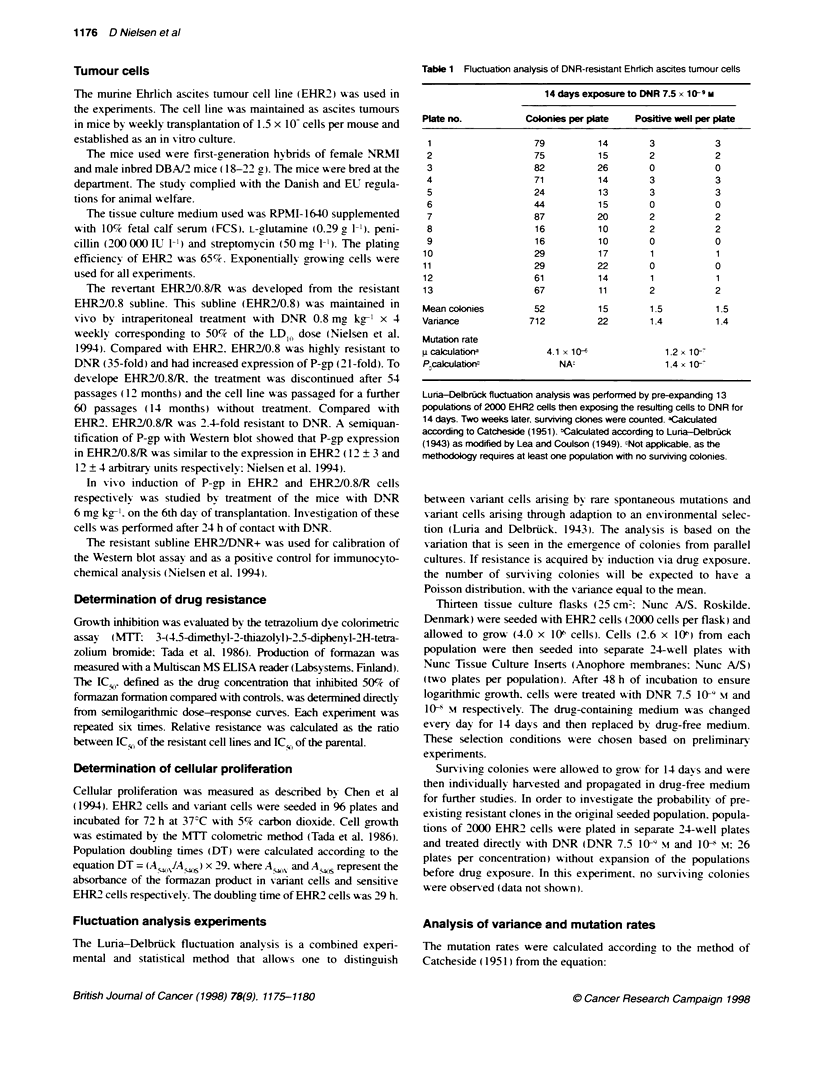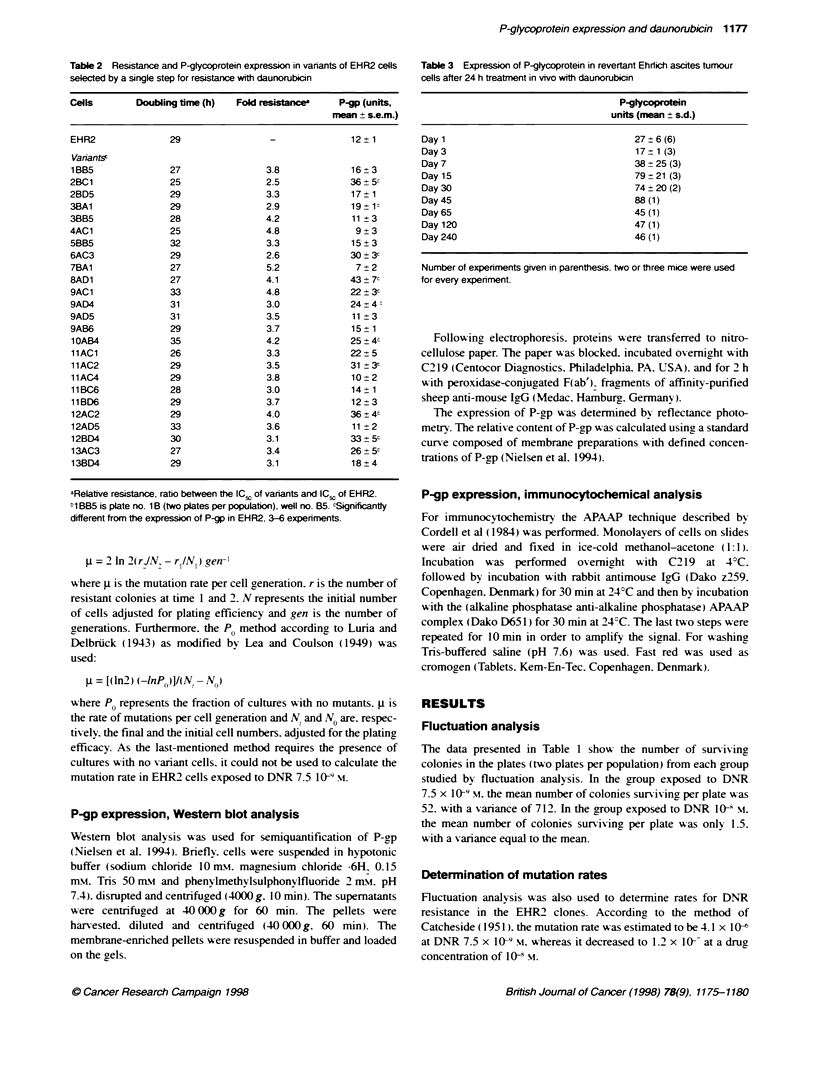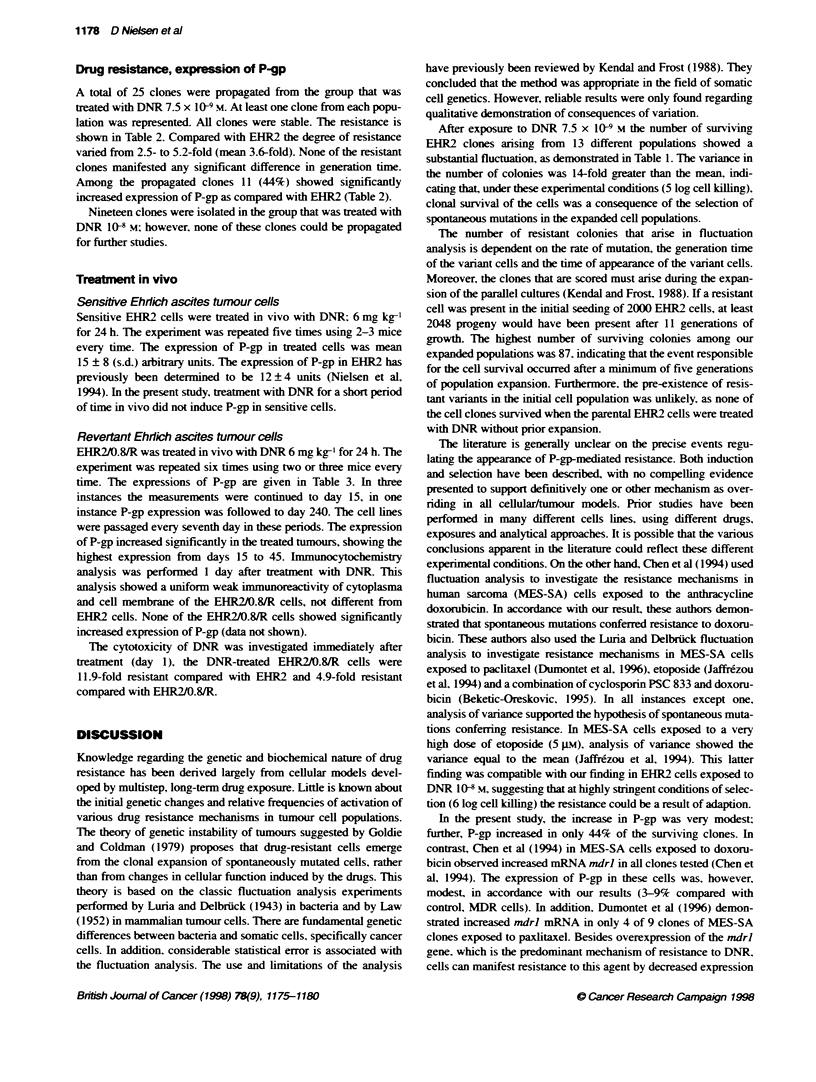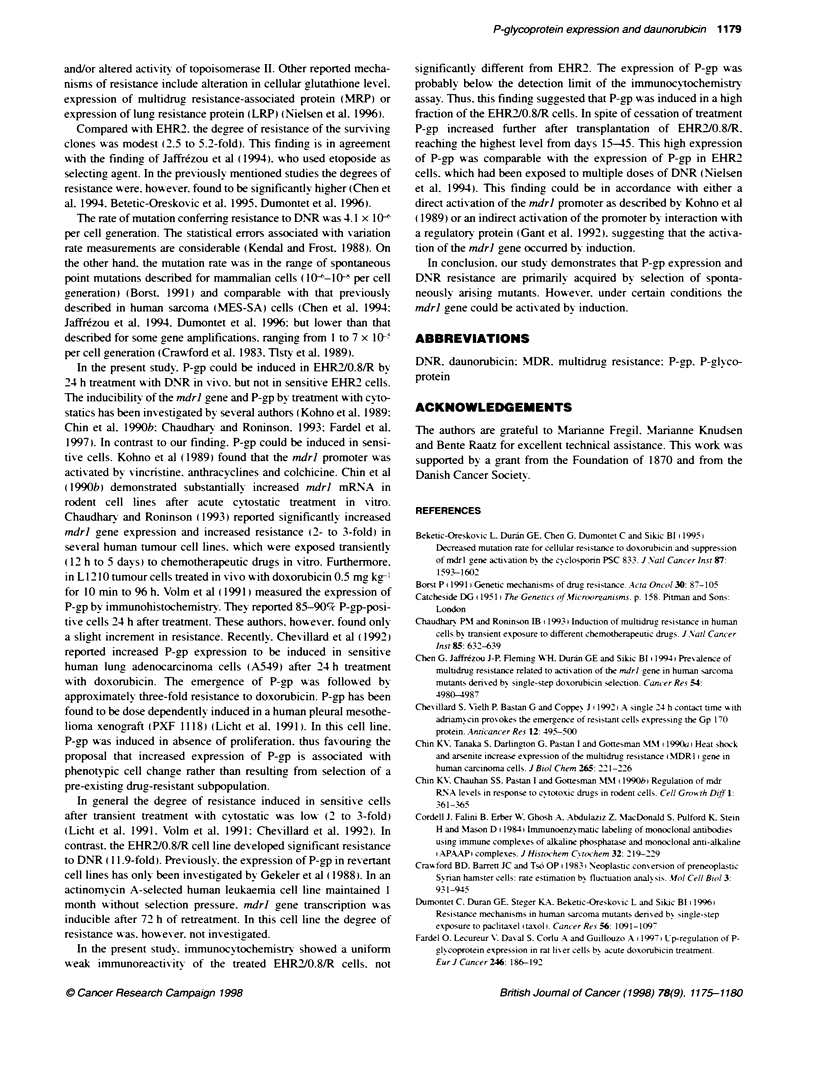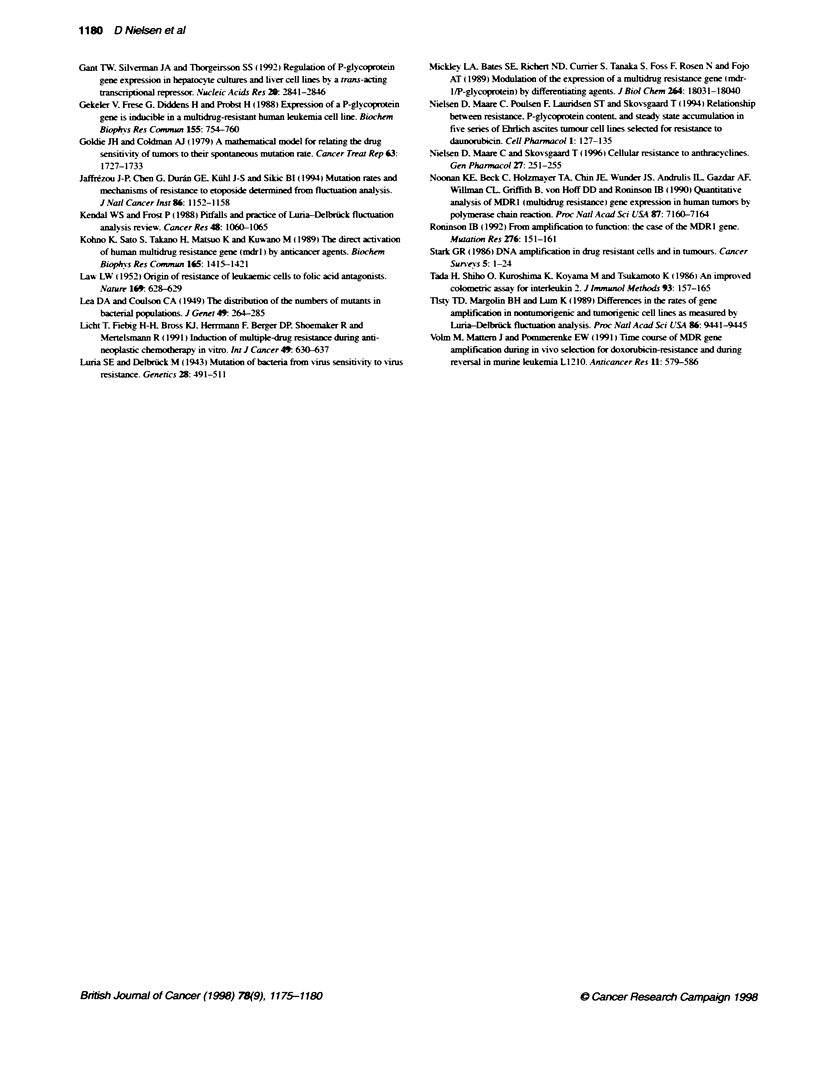# P-glycoprotein expression in Ehrlich ascites tumour cells after in vitro and in vivo selection with daunorubicin.

**DOI:** 10.1038/bjc.1998.650

**Published:** 1998-11

**Authors:** D. Nielsen, J. Eriksen, C. Maare, A. H. Jakobsen, T. Skovsgaard

**Affiliations:** Department of Oncology, Herlev Hospital, University of Copenhagen, Denmark.

## Abstract

Fluctuation analysis experiments were performed to assess whether selection or induction determines expression of P-glycoprotein and resistance in the murine Ehrlich ascites tumour cell line (EHR2) after exposure to daunorubicin. Thirteen expanded populations of EHR2 cells were exposed to daunorubicin 7.5 x 10(-9) M or 10(-8) M for 2 weeks. Surviving clones were scored and propagated. Only clones exposed to daunorubicin 7.5 x 10(-9) M could be expanded for investigation. Drug resistance was assessed by the tetrazolium dye (MTT) cytotoxicity assay. Western blot was used for determination of P-glycoprotein. Compared with EHR2, the variant cells were 2.5- to 5.2-fold resistant to daunorubicin (mean 3.6-fold). P-glycoprotein was significantly increased in 11 of 25 clones (44%). Analysis of variance supported the hypothesis that spontaneous mutations conferred drug resistance in EHR2 cells exposed to daunorubicin 7.5 x 10(-9) M. At this level (5 log cell killing) of drug exposure, the mutation rate was estimated at 4.1 x 10(-6) per cell generation. In contrast, induction seemed to determine resistance in EHR2 cells in vitro exposed to daunorubicin 10(-8) M. The revertant EHR2/0.8/R was treated in vivo with daunorubicin for 24 h. After treatment, P-glycoprotein increased in EHR2/0.8/R (sevenfold) and the cell line developed resistance to daunorubicin (12-fold), suggesting that in EHR2/0.8/R the mdr1 gene was activated by induction. In conclusion, our study demonstrates that P-glycoprotein expression and daunorubicin resistance are primarily acquired by selection of spontaneously arising mutants. However, under certain conditions the mdr1 gene may be activated by induction.


British Journal of Cancer (1998) 78(9), 1175–1180
© 1998 Cancer Research Campaign

### P-glycoprotein expression in Ehrlich ascites tumour cells after in vitro and in vivo selection with daunorubicin

**D Nielsen[1], J Eriksen[1], C Maare[1], AH Jakobsen[2] and T Skovsgaard[1]**

[1]Department of Oncology, Herlev Hospital, University of Copenhagen, Denmark; [2]Department of Haematology, Herlev Hospital, University of Copenhagen, Denmark

**Summary** Fluctuation analysis experiments were performed to assess whether selection or induction determines expression of P-glycoprotein and resistance in the murine Ehrlich ascites tumour cell line (EHR2) after exposure to daunorubicin. Thirteen expanded populations of EHR2 cells were exposed to daunorubicin $7.5 \times 10^{-9}$ M or $10^{-8}$ M for 2 weeks. Surviving clones were scored and propagated. Only clones exposed to daunorubicin $7.5 \times 10^{-9}$ M could be expanded for investigation. Drug resistance was assessed by the tetrazolium dye (MTT) cytotoxicity assay. Western blot was used for determination of P-glycoprotein. Compared with EHR2, the variant cells were 2.5- to 5.2-fold resistant to daunorubicin (mean 3.6-fold). P-glycoprotein was significantly increased in 11 of 25 clones (44%). Analysis of variance supported the hypothesis that spontaneous mutations conferred drug resistance in EHR2 cells exposed to daunorubicin $7.5 \times 10^{-9}$ M. At this level (5 log cell killing) of drug exposure, the mutation rate was estimated at $4.1 \times 10^{-6}$ per cell generation. In contrast, induction seemed to determine resistance in EHR2 cells in vitro exposed to daunorubicin $10^{-8}$ M. The revertant EHR2/0.8/R was treated in vivo with daunorubicin for 24 h. After treatment, P-glycoprotein increased in EHR2/0.8/R (sevenfold) and the cell line developed resistance to daunorubicin (12-fold), suggesting that in EHR2/0.8/R the *mdr1* gene was activated by induction. In conclusion, our study demonstrates that P-glycoprotein expression and daunorubicin resistance are primarily acquired by selection of spontaneously arising mutants. However, under certain conditions the *mdr1* gene may be activated by induction.

**Keywords:** daunorubicin; P-glycoprotein; resistance

Repeated exposure of tumour cell lines to cytotoxic agents can lead to the development of sublines that are highly resistant to the selecting agent. In several established cell lines, amplification of a specific gene has been found to be responsible for resistance (Stark, 1986). However, selection for gene amplification is only possible when the gene is active. Silent genes require transcriptional activation before selection for amplification. The *mdr1* gene encoding the multidrug resistance (MDR) protein, P-glycoprotein (P-gp), belongs to this group of genes (Noonan et al, 1990). A number of MDR cell lines have shown increased expression of the *mdr1* gene with little, if any, gene amplification (Roninson, 1992). Increased transcription may arise by mutation (e.g. in genes coding for regulatory proteins) or induction (reviewed in Borst, 1991). Development of resistance by induction demands the continuous presence of drug, whereas drug resistance developed by mutations occurs at random in both the presence and absence of drug. Drug treatment subsequently selects the resistant cells and their progony will dominate in recurring tumours as described by Goldie and Coldman (1979).

Several reports indicate that transcription of the *mdr1* gene can be induced in cell lines. Thus, a variety of environmental stresses and circumstances that affect cellular differentiation have been shown to induce *mdr1* gene expression (Chin et al, 1990*a*; Mickley et al, 1996). The anthracycline doxorubicin has been shown to induce expression of P-gp after only 24 h of contact with sensitive cells (Volm et al, 1991; Chevillard et al, 1992). The *mdr1* promoter has been shown to respond directly to treatment with anthracyclines or vinca alkaloids (Kohno et al, 1989). On the other hand, fluctuation analysis experiments have indicated that activation of the *mdr1* gene in human sarcoma cells occurs as a result of a stochastic process consistent with mutational events (Chen et al, 1994).

The purpose of the present study was: (1) to use Luria–Delbrück fluctuation analysis to investigate whether daunorubicin (DNR) induced P-gp in sensitive Ehrlich ascites tumour cells (EHR2) by mutation or induction; (2) to investigate whether P-gp could be induced in EHR2 by 24 h treatment in vivo with DNR; (3) to investigate the expression of P-gp after 24-h treatment in vivo with DNR in cells that are retreated after reversal of resistance.

### MATERIALS AND METHODS

### Chemicals

Daunorubicin as hydrochloride was purchased from Farmitalia Carlo Erba, Milan, Italy. All other chemicals were of analytical grade.

*Received 5 December 1997*
*Revised 21 March 1998*
*Accepted 25 March 1998*

*Correspondence to:* D Nielsen, Department of Oncology 5072, Finsen Centret, Rigshospitalet, University of Copenhagen, 9 Blegdamsvej DK-2100 Copenhagen, Denmark

### Tumour cells

The murine Ehrlich ascites tumour cell line (EHR2) was used in the experiments. The cell line was maintained as ascites tumours in mice by weekly transplantation of $1.5 \times 10^7$ cells per mouse and established as an in vitro culture.

The mice used were first-generation hybrids of female NRMI and male inbred DBA/2 mice (18–22 g). The mice were bred at the department. The study complied with the Danish and EU regulations for animal welfare.

The tissue culture medium used was RPMI-1640 supplemented with 10% fetal calf serum (FCS), L-glutamine (0.29 g $l^{-1}$), penicillin (200 000 IU $l^{-1}$) and streptomycin (50 mg $l^{-1}$). The plating efficiency of EHR2 was 65%. Exponentially growing cells were used for all experiments.

The revertant EHR2/0.8/R was developed from the resistant EHR2/0.8 subline. This subline (EHR2/0.8) was maintained in vivo by intraperitoneal treatment with DNR 0.8 mg $kg^{-1} \times 4$ weekly corresponding to 50% of the $LD_{10}$ dose (Nielsen et al, 1994). Compared with EHR2, EHR2/0.8 was highly resistant to DNR (35-fold) and had increased expression of P-gp (21-fold). To develope EHR2/0.8/R, the treatment was discontinued after 54 passages (12 months) and the cell line was passaged for a further 60 passages (14 months) without treatment. Compared with EHR2, EHR2/0.8/R was 2.4-fold resistant to DNR. A semiquantification of P-gp with Western blot showed that P-gp expression in EHR2/0.8/R was similar to the expression in EHR2 (12 ± 3 and 12 ± 4 arbitrary units respectively; Nielsen et al, 1994).

In vivo induction of P-gp in EHR2 and EHR2/0.8/R cells respectively was studied by treatment of the mice with DNR 6 mg $kg^{-1}$, on the 6th day of transplantation. Investigation of these cells was performed after 24 h of contact with DNR.

The resistant subline EHR2/DNR+ was used for calibration of the Western blot assay and as a positive control for immunocytochemical analysis (Nielsen et al, 1994).

### Determination of drug resistance

Growth inhibition was evaluated by the tetrazolium dye colorimetric assay (MTT; 3-(4,5-dimethyl-2-thiazolyl)-2,5-diphenyl-2H-tetrazolium bromide; Tada et al, 1986). Production of formazan was measured with a Multiscan MS ELISA reader (Labsystems, Finland). The $IC_{50}$, defined as the drug concentration that inhibited 50% of formazan formation compared with controls, was determined directly from semilogarithmic dose–response curves. Each experiment was repeated six times. Relative resistance was calculated as the ratio between $IC_{50}$ of the resistant cell lines and $IC_{50}$ of the parental.

### Determination of cellular proliferation

Cellular proliferation was measured as described by Chen et al (1994). EHR2 cells and variant cells were seeded in 96 plates and incubated for 72 h at 37°C with 5% carbon dioxide. Cell growth was estimated by the MTT colometric method (Tada et al, 1986). Population doubling times (DT) were calculated according to the equation $DT = (A_{540V}/A_{540S}) \times 29$, where $A_{540V}$ and $A_{540S}$ represent the absorbance of the formazan product in variant cells and sensitive EHR2 cells respectively. The doubling time of EHR2 cells was 29 h.

### Fluctuation analysis experiments

The Luria–Delbrück fluctuation analysis is a combined experimental and statistical method that allows one to distinguish between variant cells arising by rare spontaneous mutations and variant cells arising through adaption to an environmental selection (Luria and Delbrück, 1943). The analysis is based on the variation that is seen in the emergence of colonies from parallel cultures. If resistance is acquired by induction via drug exposure, the number of surviving colonies will be expected to have a Poisson distribution, with the variance equal to the mean.

**Table 1** Fluctuation analysis of DNR-resistant Ehrlich ascites tumour cells

| | 14 days exposure to DNR $7.5 \times 10^{-9}$ M | | | |
|---|---|---|---|---|
| Plate no. | Colonies per plate | | Positive well per plate | |
| 1 | 79 | 14 | 3 | 3 |
| 2 | 75 | 15 | 2 | 2 |
| 3 | 82 | 26 | 0 | 0 |
| 4 | 71 | 14 | 3 | 3 |
| 5 | 24 | 13 | 3 | 3 |
| 6 | 44 | 15 | 0 | 0 |
| 7 | 87 | 20 | 2 | 2 |
| 8 | 16 | 10 | 2 | 2 |
| 9 | 16 | 10 | 0 | 0 |
| 10 | 29 | 17 | 1 | 1 |
| 11 | 29 | 22 | 0 | 0 |
| 12 | 61 | 14 | 1 | 1 |
| 13 | 67 | 11 | 2 | 2 |
| Mean colonies | 52 | 15 | 1.5 | 1.5 |
| Variance | 712 | 22 | 1.4 | 1.4 |
| Mutation rate | | | | |
| μ calculation[a] | $4.1 \times 10^{-6}$ | | $1.2 \times 10^{-7}$ | |
| $P_0$calculation[b] | NA[c] | | $1.4 \times 10^{-7}$ | |

Luria–Delbrück fluctuation analysis was performed by pre-expanding 13 populations of 2000 EHR2 cells then exposing the resulting cells to DNR for 14 days. Two weeks later, surviving clones were counted. [a]Calculated according to Catcheside (1951). [b]Calculated according to Luria–Delbrück (1943) as modified by Lea and Coulson (1949). [c]Not applicable, as the methodology requires at least one population with no surviving colonies.

Thirteen tissue culture flasks (25 $cm^2$; Nunc A/S, Roskilde, Denmark) were seeded with EHR2 cells (2000 cells per flask) and allowed to grow ($4.0 \times 10^6$ cells). Cells ($2.6 \times 10^6$) from each population were then seeded into separate 24-well plates with Nunc Tissue Culture Inserts (Anophore membranes; Nunc A/S) (two plates per population). After 48 h of incubation to ensure logarithmic growth, cells were treated with DNR 7.5 $10^{-9}$ M and $10^{-8}$ M respectively. The drug-containing medium was changed every day for 14 days and then replaced by drug-free medium. These selection conditions were chosen based on preliminary experiments.

Surviving colonies were allowed to grow for 14 days and were then individually harvested and propagated in drug-free medium for further studies. In order to investigate the probability of pre-existing resistant clones in the original seeded population, populations of 2000 EHR2 cells were plated in separate 24-well plates and treated directly with DNR (DNR 7.5 $10^{-9}$ M and $10^{-8}$ M; 26 plates per concentration) without expansion of the populations before drug exposure. In this experiment, no surviving colonies were observed (data not shown).

### Analysis of variance and mutation rates

The mutation rates were calculated according to the method of Catcheside (1951) from the equation:

© Cancer Research Campaign 1998

**Table 2** Resistance and P-glycoprotein expression in variants of EHR2 cells selected by a single step for resistance with daunorubicin

| Cells | Doubling time (h) | Fold resistance[a] | P-gp (units, mean ± s.e.m.) |
|---|---|---|---|
| EHR2 | 29 | - | 12 ± 1 |
| *Variants*[b] | | | |
| 1BB5 | 27 | 3.8 | 16 ± 3 |
| 2BC1 | 25 | 2.5 | 36 ± 5[c] |
| 2BD5 | 29 | 3.3 | 17 ± 1 |
| 3BA1 | 29 | 2.9 | 19 ± 1[c] |
| 3BB5 | 28 | 4.2 | 11 ± 3 |
| 4AC1 | 25 | 4.8 | 9 ± 3 |
| 5BB5 | 32 | 3.3 | 15 ± 3 |
| 6AC3 | 29 | 2.6 | 30 ± 3[c] |
| 7BA1 | 27 | 5.2 | 7 ± 2 |
| 8AD1 | 27 | 4.1 | 43 ± 7[c] |
| 9AC1 | 33 | 4.8 | 22 ± 3[c] |
| 9AD4 | 31 | 3.0 | 24 ± 4[c] |
| 9AD5 | 31 | 3.5 | 11 ± 3 |
| 9AB6 | 29 | 3.7 | 15 ± 1 |
| 10AB4 | 35 | 4.2 | 25 ± 4[c] |
| 11AC1 | 26 | 3.3 | 22 ± 5 |
| 11AC2 | 29 | 3.5 | 31 ± 3[c] |
| 11AC4 | 29 | 3.8 | 10 ± 2 |
| 11BC6 | 28 | 3.0 | 14 ± 1 |
| 11BD6 | 29 | 3.7 | 12 ± 3 |
| 12AC2 | 29 | 4.0 | 36 ± 4[c] |
| 12AD5 | 33 | 3.6 | 11 ± 2 |
| 12BD4 | 30 | 3.1 | 33 ± 5[c] |
| 13AC3 | 27 | 3.4 | 26 ± 5[c] |
| 13BD4 | 29 | 3.1 | 18 ± 4 |

[a]Relative resistance, ratio between the $IC_{50}$ of variants and $IC_{50}$ of EHR2. [b]1BB5 is plate no. 1B (two plates per population), well no. B5. [c]Significantly different from the expression of P-gp in EHR2, 3–6 experiments.

$$\mu = 2 \ln 2(r_2/N_2 - r_1/N_1)\, gen^{-1}$$

where μ is the mutation rate per cell generation, *r* is the number of resistant colonies at time 1 and 2, *N* represents the initial number of cells adjusted for plating efficiency and *gen* is the number of generations. Furthermore, the $P_0$ method according to Luria and Delbrück (1943) as modified by Lea and Coulson (1949) was used:

$$\mu = [(\ln 2)(-\ln P_0)]/(N_t - N_0)$$

where $P_0$ represents the fraction of cultures with no mutants, μ is the rate of mutations per cell generation and $N_t$ and $N_0$ are, respectively, the final and the initial cell numbers, adjusted for the plating efficacy. As the last-mentioned method requires the presence of cultures with no variant cells, it could not be used to calculate the mutation rate in EHR2 cells exposed to DNR 7.5 $10^{-9}$ M.

### P-gp expression, Western blot analysis

Western blot analysis was used for semiquantification of P-gp (Nielsen et al, 1994). Briefly, cells were suspended in hypotonic buffer (sodium chloride 10 mM, magnesium chloride $\cdot 6H_2$ 0.15 mM, Tris 50 mM and phenylmethylsulphonylfluoride 2 mM, pH 7.4), disrupted and centrifuged (4000 *g*, 10 min). The supernatants were centrifuged at 40 000 *g* for 60 min. The pellets were harvested, diluted and centrifuged (40 000 *g*, 60 min). The membrane-enriched pellets were resuspended in buffer and loaded on the gels.

**Table 3** Expression of P-glycoprotein in revertant Ehrlich ascites tumour cells after 24 h treatment in vivo with daunorubicin

| | P-glycoprotein units (mean ± s.d.) |
|---|---|
| Day 1 | 27 ± 6 (6) |
| Day 3 | 17 ± 1 (3) |
| Day 7 | 38 ± 25 (3) |
| Day 15 | 79 ± 21 (3) |
| Day 30 | 74 ± 20 (2) |
| Day 45 | 88 (1) |
| Day 65 | 45 (1) |
| Day 120 | 47 (1) |
| Day 240 | 46 (1) |

Number of experiments given in parenthesis, two or three mice were used for every experiment.

Following electrophoresis, proteins were transferred to nitrocellulose paper. The paper was blocked, incubated overnight with C219 (Centocor Diagnostics, Philadelphia, PA, USA), and for 2 h with peroxidase-conjugated $F(ab')_2$ fragments of affinity-purified sheep anti-mouse IgG (Medac, Hamburg, Germany).

The expression of P-gp was determined by reflectance photometry. The relative content of P-gp was calculated using a standard curve composed of membrane preparations with defined concentrations of P-gp (Nielsen et al, 1994).

### P-gp expression, immunocytochemical analysis

For immunocytochemistry the APAAP technique described by Cordell et al (1984) was performed. Monolayers of cells on slides were air dried and fixed in ice-cold methanol–acetone (1:1). Incubation was performed overnight with C219 at 4°C, followed by incubation with rabbit antimouse IgG (Dako z259, Copenhagen, Denmark) for 30 min at 24°C and then by incubation with the (alkaline phosphatase anti-alkaline phosphatase) APAAP complex (Dako D651) for 30 min at 24°C. The last two steps were repeated for 10 min in order to amplify the signal. For washing Tris-buffered saline (pH 7.6) was used. Fast red was used as cromogen (Tablets, Kem-En-Tec, Copenhagen, Denmark).

### RESULTS

### Fluctuation analysis

The data presented in Table 1 show the number of surviving colonies in the plates (two plates per population) from each group studied by fluctuation analysis. In the group exposed to DNR $7.5 \times 10^{-9}$ M, the mean number of colonies surviving per plate was 52, with a variance of 712. In the group exposed to DNR $10^{-8}$ M, the mean number of colonies surviving per plate was only 1.5, with a variance equal to the mean.

### Determination of mutation rates

Fluctuation analysis was also used to determine rates for DNR resistance in the EHR2 clones. According to the method of Catcheside (1951), the mutation rate was estimated to be $4.1 \times 10^{-6}$ at DNR $7.5 \times 10^{-9}$ M, whereas it decreased to $1.2 \times 10^{-7}$ at a drug concentration of $10^{-8}$ M.

### Drug resistance, expression of P-gp

A total of 25 clones were propagated from the group that was treated with DNR 7.5 × $10^{-9}$ M. At least one clone from each population was represented. All clones were stable. The resistance is shown in Table 2. Compared with EHR2 the degree of resistance varied from 2.5- to 5.2-fold (mean 3.6-fold). None of the resistant clones manifested any significant difference in generation time. Among the propagated clones 11 (44%) showed significantly increased expression of P-gp as compared with EHR2 (Table 2).

Nineteen clones were isolated in the group that was treated with DNR $10^{-8}$ M; however, none of these clones could be propagated for further studies.

### Treatment in vivo

*Sensitive Ehrlich ascites tumour cells*

Sensitive EHR2 cells were treated in vivo with DNR; 6 mg $kg^{-1}$ for 24 h. The experiment was repeated five times using 2–3 mice every time. The expression of P-gp in treated cells was mean 15 ± 8 (s.d.) arbitrary units. The expression of P-gp in EHR2 has previously been determined to be 12 ± 4 units (Nielsen et al, 1994). In the present study, treatment with DNR for a short period of time in vivo did not induce P-gp in sensitive cells.

*Revertant Ehrlich ascites tumour cells*

EHR2/0.8/R was treated in vivo with DNR 6 mg $kg^{-1}$ for 24 h. The experiment was repeated six times using two or three mice every time. The expressions of P-gp are given in Table 3. In three instances the measurements were continued to day 15, in one instance P-gp expression was followed to day 240. The cell lines were passaged every seventh day in these periods. The expression of P-gp increased significantly in the treated tumours, showing the highest expression from days 15 to 45. Immunocytochemistry analysis was performed 1 day after treatment with DNR. This analysis showed a uniform weak immunoreactivity of cytoplasma and cell membrane of the EHR2/0.8/R cells, not different from EHR2 cells. None of the EHR2/0.8/R cells showed significantly increased expression of P-gp (data not shown).

The cytotoxicity of DNR was investigated immediately after treatment (day 1), the DNR-treated EHR2/0.8/R cells were 11.9-fold resistant compared with EHR2 and 4.9-fold resistant compared with EHR2/0.8/R.

### DISCUSSION

Knowledge regarding the genetic and biochemical nature of drug resistance has been derived largely from cellular models developed by multistep, long-term drug exposure. Little is known about the initial genetic changes and relative frequencies of activation of various drug resistance mechanisms in tumour cell populations. The theory of genetic instability of tumours suggested by Goldie and Coldman (1979) proposes that drug-resistant cells emerge from the clonal expansion of spontaneously mutated cells, rather than from changes in cellular function induced by the drugs. This theory is based on the classic fluctuation analysis experiments performed by Luria and Delbrück (1943) in bacteria and by Law (1952) in mammalian tumour cells. There are fundamental genetic differences between bacteria and somatic cells, specifically cancer cells. In addition, considerable statistical error is associated with the fluctuation analysis. The use and limitations of the analysis have previously been reviewed by Kendal and Frost (1988). They concluded that the method was appropriate in the field of somatic cell genetics. However, reliable results were only found regarding qualitative demonstration of consequences of variation.

After exposure to DNR 7.5 × $10^{-9}$ M the number of surviving EHR2 clones arising from 13 different populations showed a substantial fluctuation, as demonstrated in Table 1. The variance in the number of colonies was 14-fold greater than the mean, indicating that, under these experimental conditions (5 log cell killing), clonal survival of the cells was a consequence of the selection of spontaneous mutations in the expanded cell populations.

The number of resistant colonies that arise in fluctuation analysis is dependent on the rate of mutation, the generation time of the variant cells and the time of appearance of the variant cells. Moreover, the clones that are scored must arise during the expansion of the parallel cultures (Kendal and Frost, 1988). If a resistant cell was present in the initial seeding of 2000 EHR2 cells, at least 2048 progeny would have been present after 11 generations of growth. The highest number of surviving colonies among our expanded populations was 87, indicating that the event responsible for the cell survival occurred after a minimum of five generations of population expansion. Furthermore, the pre-existence of resistant variants in the initial cell population was unlikely, as none of the cell clones survived when the parental EHR2 cells were treated with DNR without prior expansion.

The literature is generally unclear on the precise events regulating the appearance of P-gp-mediated resistance. Both induction and selection have been described, with no compelling evidence presented to support definitively one or other mechanism as overriding in all cellular/tumour models. Prior studies have been performed in many different cells lines, using different drugs, exposures and analytical approaches. It is possible that the various conclusions apparent in the literature could reflect these different experimental conditions. On the other hand, Chen et al (1994) used fluctuation analysis to investigate the resistance mechanisms in human sarcoma (MES-SA) cells exposed to the anthracycline doxorubicin. In accordance with our result, these authors demonstrated that spontaneous mutations conferred resistance to doxorubicin. These authors also used the Luria and Delbrück fluctuation analysis to investigate resistance mechanisms in MES-SA cells exposed to paclitaxel (Dumontet et al, 1996), etoposide (Jaffrézou et al, 1994) and a combination of cyclosporin PSC 833 and doxorubicin (Beketic-Oreskovic, 1995). In all instances except one, analysis of variance supported the hypothesis of spontaneous mutations conferring resistance. In MES-SA cells exposed to a very high dose of etoposide (5 μM), analysis of variance showed the variance equal to the mean (Jaffrézou et al, 1994). This latter finding was compatible with our finding in EHR2 cells exposed to DNR $10^{-8}$ M, suggesting that at highly stringent conditions of selection (6 log cell killing) the resistance could be a result of adaption.

In the present study, the increase in P-gp was very modest; further, P-gp increased in only 44% of the surviving clones. In contrast, Chen et al (1994) in MES-SA cells exposed to doxorubicin observed increased mRNA *mdr1* in all clones tested (Chen et al, 1994). The expression of P-gp in these cells was, however, modest, in accordance with our results (3–9% compared with control, MDR cells). In addition, Dumontet et al (1996) demonstrated increased *mdr1* mRNA in only 4 of 9 clones of MES-SA clones exposed to paxlitaxel. Besides overexpression of the *mdr1* gene, which is the predominant mechanism of resistance to DNR, cells can manifest resistance to this agent by decreased expression

© Cancer Research Campaign 1998

and/or altered activity of topoisomerase II. Other reported mechanisms of resistance include alteration in cellular glutathione level, expression of multidrug resistance-associated protein (MRP) or expression of lung resistance protein (LRP) (Nielsen et al, 1996).

Compared with EHR2, the degree of resistance of the surviving clones was modest (2.5 to 5.2-fold). This finding is in agreement with the finding of Jaffrézou et al (1994), who used etoposide as selecting agent. In the previously mentioned studies the degrees of resistance were, however, found to be significantly higher (Chen et al, 1994, Betetic-Oreskovic et al, 1995, Dumontet et al, 1996).

The rate of mutation conferring resistance to DNR was $4.1 \times 10^{-6}$ per cell generation. The statistical errors associated with variation rate measurements are considerable (Kendal and Frost, 1988). On the other hand, the mutation rate was in the range of spontaneous point mutations described for mammalian cells ($10^{-6}$–$10^{-8}$ per cell generation) (Borst, 1991) and comparable with that previously described in human sarcoma (MES-SA) cells (Chen et al, 1994; Jaffrézou et al, 1994, Dumontet et al, 1996; but lower than that described for some gene amplifications, ranging from 1 to $7 \times 10^{-5}$ per cell generation (Crawford et al, 1983, Tlsty et al, 1989).

In the present study, P-gp could be induced in EHR2/0.8/R by 24 h treatment with DNR in vivo, but not in sensitive EHR2 cells. The inducibility of the *mdr1* gene and P-gp by treatment with cytostatics has been investigated by several authors (Kohno et al, 1989; Chin et al, 1990*b*; Chaudhary and Roninson, 1993; Fardel et al, 1997). In contrast to our finding, P-gp could be induced in sensitive cells. Kohno et al (1989) found that the *mdr1* promoter was activated by vincristine, anthracyclines and colchicine. Chin et al (1990*b*) demonstrated substantially increased *mdr1* mRNA in rodent cell lines after acute cytostatic treatment in vitro. Chaudhary and Roninson (1993) reported significantly increased *mdr1* gene expression and increased resistance (2- to 3-fold) in several human tumour cell lines, which were exposed transiently (12 h to 5 days) to chemotherapeutic drugs in vitro. Furthermore, in L1210 tumour cells treated in vivo with doxorubicin 0.5 mg $kg^{-1}$ for 10 min to 96 h, Volm et al (1991) measured the expression of P-gp by immunohistochemistry. They reported 85–90% P-gp-positive cells 24 h after treatment. These authors, however, found only a slight increment in resistance. Recently, Chevillard et al (1992) reported increased P-gp expression to be induced in sensitive human lung adenocarcinoma cells (A549) after 24 h treatment with doxorubicin. The emergence of P-gp was followed by approximately three-fold resistance to doxorubicin. P-gp has been found to be dose dependently induced in a human pleural mesothelioma xenograft (PXF 1118) (Licht et al, 1991). In this cell line, P-gp was induced in absence of proliferation, thus favouring the proposal that increased expression of P-gp is associated with phenotypic cell change rather than resulting from selection of a pre-existing drug-resistant subpopulation.

In general the degree of resistance induced in sensitive cells after transient treatment with cytostatic was low (2 to 3-fold) (Licht et al, 1991, Volm et al, 1991; Chevillard et al, 1992). In contrast, the EHR2/0.8/R cell line developed significant resistance to DNR (11.9-fold). Previously, the expression of P-gp in revertant cell lines has only been investigated by Gekeler et al (1988). In an actinomycin A-selected human leukaemia cell line maintained 1 month without selection pressure, *mdr1* gene transcription was inducible after 72 h of retreatment. In this cell line the degree of resistance was, however, not investigated.

In the present study, immunocytochemistry showed a uniform weak immunoreactivity of the treated EHR2/0.8/R cells, not significantly different from EHR2. The expression of P-gp was probably below the detection limit of the immunocytochemistry assay. Thus, this finding suggested that P-gp was induced in a high fraction of the EHR2/0.8/R cells. In spite of cessation of treatment P-gp increased further after transplantation of EHR2/0.8/R, reaching the highest level from days 15–45. This high expression of P-gp was comparable with the expression of P-gp in EHR2 cells, which had been exposed to multiple doses of DNR (Nielsen et al, 1994). This finding could be in accordance with either a direct activation of the *mdr1* promoter as described by Kohno et al (1989) or an indirect activation of the promoter by interaction with a regulatory protein (Gant et al, 1992), suggesting that the activation of the *mdr1* gene occurred by induction.

In conclusion, our study demonstrates that P-gp expression and DNR resistance are primarily acquired by selection of spontaneously arising mutants. However, under certain conditions the *mdr1* gene could be activated by induction.

### ABBREVIATIONS

DNR, daunorubicin; MDR, multidrug resistance; P-gp, P-glycoprotein

### ACKNOWLEDGEMENTS

The authors are grateful to Marianne Fregil, Marianne Knudsen and Bente Raatz for excellent technical assistance. This work was supported by a grant from the Foundation of 1870 and from the Danish Cancer Society.

### REFERENCES

Beketic-Oreskovic L, Durán GE, Chen G, Dumontet C and Sikic BI (1995) Decreased mutation rate for cellular resistance to doxorubicin and suppression of mdr1 gene activation by the cyclosporin PSC 833. *J Natl Cancer Inst* **87**: 1593–1602

Borst P (1991) Genetic mechanisms of drug resistance. *Acta Oncol* **30**: 87–105

Catcheside DG (1951) *The Genetics of Microorganisms*, p. 158. Pitman and Sons: London

Chaudhary PM and Roninson IB (1993) Induction of multidrug resistance in human cells by transient exposure to different chemotherapeutic drugs. *J Natl Cancer Inst* **85**: 632–639

Chen G, Jaffrézou J-P, Fleming WH, Durán GE and Sikic BI (1994) Prevalence of multidrug resistance related to activation of the *mdr1* gene in human sarcoma mutants derived by single-step doxorubicin selection. *Cancer Res* **54**: 4980–4987

Chevillard S, Vielh P, Bastan G and Coppey J (1992) A single 24 h contact time with adriamycin provokes the emergence of resistant cells expressing the Gp 170 protein. *Anticancer Res* **12**: 495–500

Chin KV, Tanaka S, Darlington G, Pastan I and Gottesman MM (1990*a*) Heat shock and arsenite increase expression of the multidrug resistance (MDR1) gene in human carcinoma cells. *J Biol Chem* **265**: 221–226

Chin KV, Chauhan SS, Pastan I and Gottesman MM (1990*b*) Regulation of mdr RNA levels in response to cytotoxic drugs in rodent cells. *Cell Growth Diff* **1**: 361–365

Cordell J, Falini B, Erber W, Ghosh A, Abdulaziz Z, MacDonald S, Pulford K, Stein H and Mason D (1984) Immunoenzymatic labeling of monoclonal antibodies using immune complexes of alkaline phosphatase and monoclonal anti-alkaline (APAAP) complexes. *J Histochem Cytochem* **32**: 219–229

Crawford BD, Barrett JC and Tsó OP (1983) Neoplastic conversion of preneoplastic Syrian hamster cells: rate estimation by fluctuation analysis. *Mol Cell Biol* **3**: 931–945

Dumontet C, Duran GE, Steger KA, Beketic-Oreskovic L and Sikic BI (1996) Resistance mechanisms in human sarcoma mutants derived by single-step exposure to paclitaxel (taxol). *Cancer Res* **56**: 1091–1097

Fardel O, Lecureur V, Daval S, Corlu A and Guillouzo A (1997) Up-regulation of P-glycoprotein expression in rat liver cells by acute doxorubicin treatment. *Eur J Cancer* **246**: 186–192

Gant TW. Silverman JA and Thorgeirsson SS (1992) Regulation of P-glycoprotein gene expression in hepatocyte cultures and liver cell lines by a *trans*-acting transcriptional repressor. *Nucleic Acids Res* **20**: 2841–2846

Gekeler V. Frese G. Diddens H and Probst H (1988) Expression of a P-glycoprotein gene is inducible in a multidrug-resistant human leukemia cell line. *Biochem Biophys Res Commun* **155**: 754–760

Goldie JH and Coldman AJ (1979) A mathematical model for relating the drug sensitivity of tumors to their spontaneous mutation rate. *Cancer Treat Rep* **63**: 1727–1733

Jaffrézou J-P. Chen G. Durán GE. Kühl J-S and Sikic BI (1994) Mutation rates and mechanisms of resistance to etoposide determined from fluctuation analysis. *J Natl Cancer Inst* **86**: 1152–1158

Kendal WS and Frost P (1988) Pitfalls and practice of Luria–Delbrück fluctuation analysis review. *Cancer Res* **48**: 1060–1065

Kohno K. Sato S. Takano H. Matsuo K and Kuwano M (1989) The direct activation of human multidrug resistance gene (mdr1) by anticancer agents. *Biochem Biophys Res Commun* **165**: 1415–1421

Law LW (1952) Origin of resistance of leukaemic cells to folic acid antagonists. *Nature* **169**: 628–629

Lea DA and Coulson CA (1949) The distribution of the numbers of mutants in bacterial populations. *J Genet* **49**: 264–285

Licht T. Fiebig H-H. Bross KJ. Herrmann F. Berger DP. Shoemaker R and Mertelsmann R (1991) Induction of multiple-drug resistance during anti-neoplastic chemotherapy in vitro. *Int J Cancer* **49**: 630–637

Luria SE and Delbrück M (1943) Mutation of bacteria from virus sensitivity to virus resistance. *Genetics* **28**: 491–511

Mickley LA. Bates SE. Richert ND. Currier S. Tanaka S. Foss F. Rosen N and Fojo AT (1989) Modulation of the expression of a multidrug resistance gene (mdr-1/P-glycoprotein) by differentiating agents. *J Biol Chem* **264**: 18031–18040

Nielsen D. Maare C. Poulsen F. Lauridsen ST and Skovsgaard T (1994) Relationship between resistance. P-glycoprotein content. and steady state accumulation in five series of Ehrlich ascites tumour cell lines selected for resistance to daunorubicin. *Cell Pharmacol* **1**: 127–135

Nielsen D. Maare C and Skovsgaard T (1996) Cellular resistance to anthracyclines. *Gen Pharmacol* **27**: 251–255

Noonan KE. Beck C. Holzmayer TA. Chin JE. Wunder JS. Andrulis IL. Gazdar AF. Willman CL. Griffith B. von Hoff DD and Roninson IB (1990) Quantitative analysis of MDR1 (multidrug resistance) gene expression in human tumors by polymerase chain reaction. *Proc Natl Acad Sci USA* **87**: 7160–7164

Roninson IB (1992) From amplification to function: the case of the MDR1 gene. *Mutation Res* **276**: 151–161

Stark GR (1986) DNA amplification in drug resistant cells and in tumours. *Cancer Surveys* **5**: 1–24

Tada H. Shiho O. Kuroshima K. Koyama M and Tsukamoto K (1986) An improved colometric assay for interleukin 2. *J Immunol Methods* **93**: 157–165

Tlsty TD. Margolin BH and Lum K (1989) Differences in the rates of gene amplification in nontumorigenic and tumorigenic cell lines as measured by Luria–Delbrück fluctuation analysis. *Proc Natl Acad Sci USA* **86**: 9441–9445

Volm M. Mattern J and Pommerenke EW (1991) Time course of MDR gene amplification during in vivo selection for doxorubicin-resistance and during reversal in murine leukemia L1210. *Anticancer Res* **11**: 579–586

© Cancer Research Campaign 1998